# Lower *FOXP3* mRNA Expression in First-Trimester Decidual Tissue from Uncomplicated Term Pregnancies with a Male Fetus

**DOI:** 10.1155/2018/1950879

**Published:** 2018-05-29

**Authors:** Tom E. C. Kieffer, Anne Laskewitz, Marijke M. Faas, Sicco A. Scherjon, Jan Jaap H. M. Erwich, Sanne J. Gordijn, Jelmer R. Prins

**Affiliations:** ^1^Department of Obstetrics and Gynecology, University Medical Center Groningen, University of Groningen, P.O. Box 30001, 9700 RB Groningen, Netherlands; ^2^Department of Pathology and Medical Biology, Division of Medical Biology, University Medical Center Groningen, University of Groningen, P.O. Box 30001, 9700 RB Groningen, Netherlands

## Abstract

Pregnancies with a male fetus are associated with higher risks of pregnancy complications through maladaptation of the maternal immune system. The pathophysiology of this phenomenon is unknown. A possible pathway could be a fetal sex-dependent maternal immune response, since males have a Y chromosome encoding specific allogenic proteins, possibly contributing to a different response and higher complication risks. To analyze whether fetal sex affects mRNA expression of maternal immune genes in early pregnancy, real-time PCR quantification was performed in the decidual tissue from primigravid pregnancies (*n* = 20) between 10 and 12 weeks with uncomplicated term outcomes. Early-pregnancy decidual mRNA expression of the regulatory T-cell marker, *FOXP3*, was sixfold lower (*p* < 0.01) in pregnancies with a male fetus compared to pregnancies with a female fetus. Additionally, mRNA expression of *IFNγ* was sixfold (*p* < 0.05) lower in pregnancies with a male fetus. The present data imply maternal immunologic differences between pregnancies with male and female fetuses which could be involved in different pregnancy pathophysiologic outcomes. Moreover, this study indicates that researchers in reproductive immunology should always consider fetal sex bias.

## 1. Introduction

Pregnancies with a male fetus have a higher incidence of preterm birth, gestational diabetes mellitus, and preeclampsia [1–4]. The pathophysiology of this phenomenon is unknown. Since these complications of pregnancy are associated with maladaptation of the maternal immune system [5], a possible pathway could lie in a fetal sex-dependent maternal immune response. An explanation might be found in the Y chromosome in males which encodes specific allogenic proteins, possibly contributing to a different maternal immune response when a male fetus is carried.

A number of studies have shown that the maternal immune system develops a fetus-specific immune response [6–8]. Moreover, studies demonstrated fetal sex-specific cytokine levels in maternal peripheral blood during and after pregnancy [9–11]. Additionally, fetal sex was found to affect cytokine expression in placental tissue in asthmatic pregnant women [12, 13]. Until now, differences in the maternal immune response between pregnancies with male or female fetuses were only shown in the peripheral blood and postpartum placental tissue. Whether a fetal sex-specific immune response is elicited at the fetal-maternal interface already in early pregnancy is unknown.

T-regulatory cells (Tregs) are of particular interest in complicated as well as uncomplicated pregnancies because of their immunosuppressive properties [14]. Tregs skew the proinflammatory T-helper 1 response to the more tolerating T-helper 2 response cells [14]. Adequate function and optimal numbers of Tregs are essential for normal implantation and pregnancy outcome, and a lack of adequate Treg numbers is associated with adverse pregnancy outcomes such as preeclampsia, implantation failure, and infertility [14–16]. The transcription factor forkhead box protein P3 (FOXP3) has been identified as the immunosuppressive protein and marker for Tregs [17]. Whether modulation of the Treg population is dependent on fetal sex and whether Tregs play a role in the etiology of higher rates of pregnancy complications in pregnancies with a male fetus is unknown.

Besides Tregs, other immune cells such as macrophages contribute to tolerance in early pregnancy by shifting towards a more tolerating M2 phenotype and by releasing cytokines which contribute to implantation and tissue remodeling [18, 19]. Fetal sex-specific differences in activation and cytokine profiles of macrophages in placental tissue were found in a mouse study [20]. Early-pregnancy alterations in mRNA expression of macrophage-associated genes in pregnancies with complicated outcomes were demonstrated [21]. However, to our knowledge, neither human studies nor early-pregnancy studies concerning sex-specific macrophages have been performed.

T-lymphocytes and macrophages secrete cytokines that contribute to either a proinflammatory or an anti-inflammatory environment. Both pro- and anti-inflammatory cytokines play a role in implantation, placentation, and pregnancy success [22]. Whereas IL1b and interferon-*γ* (IFN*ɣ)* secretion at the fetal-maternal interface seems beneficial for successful implantation [23], increased levels of IL6 at term are associated with preterm delivery and neonatal morbidity [24, 25]. Presumably, the timing and amount of secretion determine whether a cytokine at a certain stage of pregnancy is beneficial for pregnancy maintenance.

The aim of this study is to analyze fetal sex-dependent differences in mRNA expression of maternal *FOXP3*, macrophage, and other immune-associated gene parameters at the fetal-maternal interface in early pregnancies that developed uneventfully. Hence, the unique first-trimester decidual tissue from ongoing human pregnancies with known uncomplicated term outcomes was studied.

## 2. Methods

First-trimester decidual tissue was obtained from surplus tissue at vaginally sampled chorionic villus sampling (CVS), between 10 and 12 weeks of gestation for maternal age (over 36 years of age at 18 weeks of gestation) screening for related risk of aneuploidy following the protocol from Huisman et al. [26]. Karyotype analysis was performed for all samples, and the karyogram appeared normal for all fetuses. Immediately after sampling, the decidual tissue was microscopically separated from the villi to minimize trophoblast contamination. Subsequently, samples were stored until further analysis following the protocol from Huisman et al. [26].

Patients were informed that otherwise discarded material could be used for research according to the “Guideline Good Use” by the FMWV committee (Federation of Medical Scientific Associations). Follow-up of pregnancies was available by questionnaires postpartum. Patients on medication, with a history of smoking, diabetes mellitus, or other comorbidities were excluded from the study.

Decidual tissues from 20 uncomplicated primiparous pregnancies were randomly selected (10 boys and 10 girls) (see Table 1). All women participating in this study were truly primigravid, did not undergo assisted reproductive techniques, and did not take any medication apart from folic acid. Based on NanoDrop quantity analysis, 4 samples were excluded (2 boys, 2 girls). RNA was isolated and purified; QIAzol lysis reagent (Qiagen, USA) was added, and samples were homogenized using a TissueLyser (Qiagen) (2 minutes, 50 Hertz). Thereafter, RNA was isolated using RNAeasy plus mini-kit (Qiagen). cDNA was reverse transcribed using Superscript-II Reverse Transcriptase kit (Invitrogen, USA). Three housekeeper genes (HPRT, GAPDH, and ACTB) were analyzed. HPRT was the most consistent in all samples and was therefore used for analysis. mRNA expression of *TBX21* (T-helper 1 (Th1) response), *GATA3* (T-helper 2 (Th2) response), *RORC* (T-helper 17 (Th17 response), *FOXP3* (Treg marker), *Interleukin 6 (IL6)*, *IL1b*, *interferon-γ (IFNɣ)*, *CD68* (macrophage), *IRF5* (M1 macrophages), and *MRC1* (M2 macrophages) was analyzed using TaqMan On-Demand-Gene-Expression Assays (Thermo Fisher, USA).

PCR reactions were performed in triplicates in a volume of 10 *μ*L consisting of 14 ng RNA, Mastermix (Thermo Fisher, USA), and RNA free water. Runs were performed on a ViiA7 Real-time PCR System (Thermo Fisher, USA), and mRNA data were normalized to *HPRT* mRNA expression using 2^−ΔCt^. Undetectable cycle threshold (Ct) values (>40) were analyzed as maximum Ct value (40). Outliers were excluded using Grubb's test. For analysis, GraphPad Prism version 5.04 for Microsoft Windows (GraphPad Software, USA) was used. Differences between the groups were evaluated using Mann–Whitney *U* test with Bonferroni multiple comparison corrections. *p* values < 0.05 were considered statistically significant.

## 3. Results and Discussion

**Figure 1:**
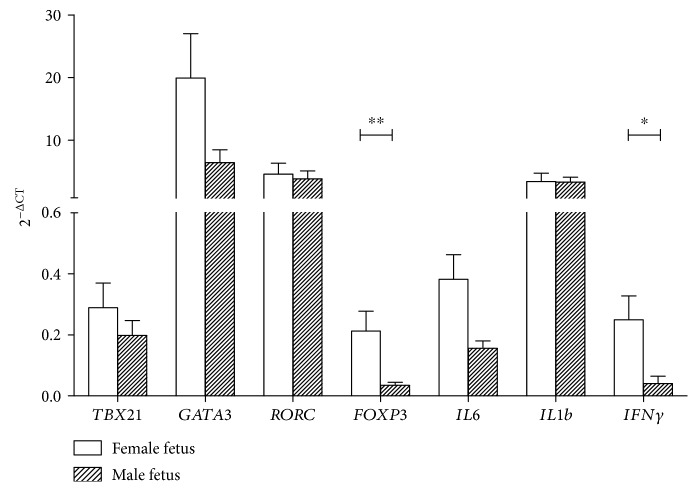
mRNA expression of T-lymphocyte markers and cytokines in the first-trimester human decidual tissue. Data are mean ± SEM mRNA target gene expression normalized to housekeeper gene *HPRT*, in the decidual tissue from pregnancies with a female fetus (open bars, *n* = 8) and pregnancies with a male fetus (black bars, *n* = 8). Comparison between groups was evaluated using Mann–Whitney *U* test with Bonferroni multiple comparison corrections; ^∗^*p* < 0.05, ^∗∗^*p* < 0.01.

In pregnancies with a male fetus, there was a sixfold significantly lower mRNA expression of *FOXP3* (*p* < 0.01) compared to pregnancies with a female fetus (see Figure 1). In both human and murine studies, it has been shown that Tregs play a role in healthy implantation and placental development in early pregnancy [27, 28]. The lower expression of *FOXP3* in the first-trimester decidual tissue from pregnancies with a male fetus could imply an inferior maternal immune tolerance in early pregnancies with a male fetus possibly contributing to a higher risk of pregnancy complications [14].

An explanation for the fetal sex-specific difference in mRNA expression of *FOXP3* could be the presence of the Y chromosome in males, which encodes minor histocompatibility antigens (HY) [29]. HY antigens are expressed in the first-trimester placental tissue and can be recognized by maternal T-lymphocytes eliciting an HY-specific immune response [11, 30, 31]. Only a limited number of studies are performed on the ability of fetal HY antigens to induce or suppress maternal *FOXP3* mRNA expression and Treg cells [32, 33]. Kahn et al. showed that HY induces an HY-specific Treg population that contributes to tolerance in mice; however, no comparison with HY absent pregnancies (solely female fetuses) was made. Therefore, no conclusions of the effects of fetal sex on *FOXP3* induction can be made [33]. Our results show that mRNA expression of the Treg marker *FOXP3* is affected by fetal sex; however, more research is necessary to clarify the role of HY in the difference of mRNA expression between pregnancies with a male and a female fetus.

In addition, significantly higher mRNA expression of *IFNγ* (*p* < 0.05) was found in pregnancies with a female fetus (see Figure 1). Many studies have associated increased IFN*γ* in different tissues with pregnancy complications such as preeclampsia [34]. Therefore, this finding could appear contradictory to the hypothesis in which male fetuses have a less favorable maternal immune environment in pregnancy. However, the proinflammatory cytokine, which is encoded by the *IFNγ* gene, has also been shown to be favorable for pregnancy [22, 35, 36]. Especially in early pregnancy, IFN*γ* has been demonstrated to be important [22, 35, 36]. IFN*γ* plays a role in implantation, placentation, and continuation of pregnancy [22, 35, 36]. Higher decidual IFN*γ* at term was associated with preeclampsia; however, women delivering preterm had lower IFN*γ* at midgestation compared to women delivering at term [34, 37, 38]. These data imply that IFN*γ* synthesis is beneficial in early pregnancy to midgestation and is unfavorable for pregnancy success in the third trimester [36]. Furthermore, IFN*γ* has been shown to be indispensable for the conversion of non-Treg cells into Treg cells [37, 38]. Since we found higher mRNA expression of both *IFNγ* and *FOXP3*, it could be postulated that during early pregnancy, the higher expression of *IFNγ* is necessary for a robust implantation and placentation and that the possible compensatory higher expression of *FOXP3* mRNA is necessary to dampen the effect of proinflammatory cytokines for successful pregnancy outcome. Herewith, the differences found in this study support the hypothesis that a difference in maternal immune response depending on fetal sex plays a role in the different incidences of pregnancy complications between pregnancies with a male or a female fetus. Our findings are in line with previous studies which showed altered fetal sex-specific cytokine levels in peripheral maternal blood during pregnancy and in placental tissue after delivery [9, 10, 12, 13].

**Figure 2:**
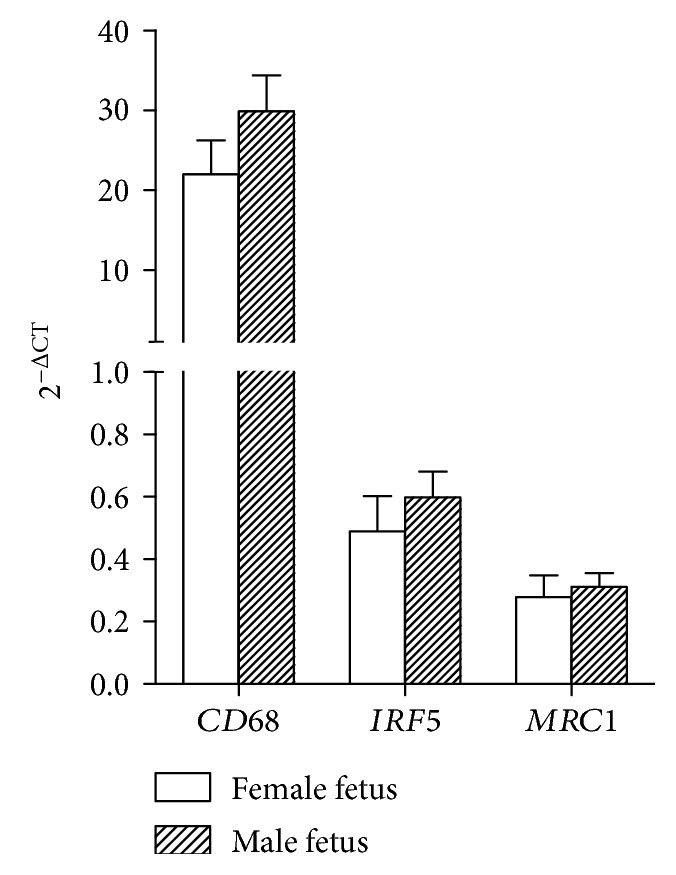
mRNA expression of macrophage markers in the first-trimester human decidual tissue. Data are mean ± SEM mRNA target gene expression normalized to housekeeper gene *HPRT*, in the decidual tissue from pregnancies with a female fetus (open bars, *n* = 8) and pregnancies with a male fetus (black bars, *n* = 8). Comparison between groups was evaluated using Mann–Whitney *U* test with Bonferroni multiple comparison corrections.

In this study, no fetal sex-specific differences in mRNA expression of macrophage markers were found (see Figure 2). Macrophage mRNA expression is possibly not influenced by fetal sex at this early stage of pregnancy, as in an obesity mouse model where a fetal sex-specific difference in macrophage activation was seen in late pregnancy but not in early-pregnancy placental tissue [20]. Or, alternatively, our sample size is too small to detect these changes.

This study uses the first-trimester decidual tissue from pregnancies with uncomplicated outcomes, which is unique and almost unobtainable. With the current knowledge on risks of pregnancy complications caused by CVS and the availability of alternative techniques [39], nowadays, CVS is not routinely performed anymore. The first-trimester decidual tissue used in this study is therefore highly appreciated and a unique possibility that enabled analysis of immune parameters in early pregnancies with known outcome. To limit the risks of pregnancy complications, the tissue volume taken with CVS was reduced to the smallest amount. The remaining tissue volume after diagnostic tests was only sufficient for PCR analysis of the genes shown and no further experiments could be performed. Therefore, actual protein synthesis and cell quantification were not investigated in this study. However, the differences in mRNA expression shown in this study do imply differences in the maternal immune response between pregnancies with a male and a female fetus. Further research is necessary to elucidate whether the different mRNA expression found does coincide with protein expression and the immune environment in early pregnancy.

In general, despite growing evidence showing the effects of fetal sex on the maternal immune response, still, most studies performed in reproductive research do not consider a fetal sex bias blurring their results. As this study shows that the maternal immune response differs depending on fetal sex, we propose that fetal sex differences between groups should always be considered.

## 4. Conclusions

In summary, this study shows fetal sex-specific differences in mRNA expression of maternal immune factors in the first-trimester decidual tissue. Lower mRNA expression of *FOXP3* and the proinflammatory cytokine encoding gene *IFNγ* was found in uncomplicated pregnancies with a male fetus compared to pregnancies with a female fetus. In the first-trimester decidual tissue studied, no differences for mRNA expression of macrophage markers were found.

These findings imply a fetal sex-dependent maternal immune response, which could be involved in the pathophysiology responsible for the higher incidence of adverse pregnancy outcomes in pregnancies with a male fetus. Moreover, this study supports that reproductive immunology research should always consider fetal sex bias.

## Figures and Tables

**Table 1:** Characteristics of patient groups.

	Pregnancies with a female fetus (*n* = 8)	Pregnancies with a male fetus (*n* = 8)
At CVS		
Maternal age (years)	37.4 ± 0.81	39.5 ± 0.46
Gestational age (weeks)	10.97 ± 0.22	10.79 ± 0.19
Gravidity	1	1
Parity	0	0
At delivery		
Gestational age (weeks)	40.5 ± 0.55	41.0 ± 0.60
Birth weight (grams)	3588 ± 120.42	3444 ± 149.41

Mean ± SEM: chorionic villus sampling; CVS: characteristics were compared between groups using Mann–Whitney *U* test with Bonferroni multiple comparison corrections.

## Data Availability

The datasets used to support this study are currently being used for further research on the topic. Access to the data will be considered upon request by contacting the corresponding author.
